# Network temperature as a metric of stability in depression symptoms across adolescence

**DOI:** 10.1038/s44220-025-00415-5

**Published:** 2025-04-29

**Authors:** Poppy Z. Grimes, Aja L. Murray, Keith Smith, Andrea G. Allegrini, Giulia G. Piazza, Henrik Larsson, Sacha Epskamp, Heather C. Whalley, Alex S. F. Kwong

**Affiliations:** 1 Division of Psychiatry, Centre for Clinical Brain Sciences, University of Edinburgh, Edinburgh, UK; 2 Department of Psychology, University of Edinburgh, Edinburgh, UK; 3 Computer and Information Sciences, University of Strathclyde, Glasgow, UK; 4 Department of Clinical, Educational and Health Psychology, Division of Psychology and Language Sciences, University College London, London, UK; 5 Social, Genetic and Developmental Psychiatry Centre, Institute of Psychiatry, Psychology and Neuroscience, King’s College London, London, UK; 6 Department of Medical Epidemiology and Biostatistics, Karolinska Institute, Stockholm, Sweden; 7 School of Medical Sciences, Örebro University, Örebro, Sweden; 8 Department of Psychology, National University of Singapore, Singapore, Singapore; 9 Generation Scotland, Centre for Genomic and Experimental Medicine, Institute of Genetics and Molecular Medicine, University of Edinburgh, Edinburgh, UK; 10 MRC Integrative Epidemiology Unit, University of Bristol, Bristol, UK

**Keywords:** Psychology, Depression

## Abstract

Depression is characterized by diverse symptom combinations that can be represented as dynamic networks. While previous research has focused on central symptoms for targeted interventions, less attention has been given to whole-network properties. Here we show that ‘network temperature’, a novel measure of psychological network stability, captures symptom alignment across adolescence—a critical period for depression onset. Network temperature reflects system stability, with higher values indicating less symptom alignment and greater variability. In three large longitudinal adolescent cohorts (total *N* = 35,901), we found that network temperature decreases across adolescence, with the steepest decline during early adolescence, particularly in males. This suggests that depression symptom networks stabilize throughout development via increased symptom alignment, potentially explaining why adolescence is a crucial period for depression onset. These findings highlight early adolescence as a key intervention window and underscore the importance of sex-specific and personalized interventions.

## Abstract (web summary)

This study explores the dynamics of adolescent depression through the lens of network temperature, a novel concept in psychological symptom networks. Researchers observed a decrease in network temperature across adolescence, indicating increased stability and decreased variability of depressive symptoms as adolescents age and with distinct patterns between sexes. This work emphasizes the importance of understanding network dynamics over static measures, offering new insights into the stability and variability of depression symptoms.

## Main

The network theory of psychopathology conceptualizes depression as emerging from the interactions among its composing symptoms^1^. Network science has provided an alternative approach to psychiatric conditions that allows us to tease apart symptom-level heterogeneity for more precise etiological understanding and intervention insights^2^. Though most studies have examined cross-sectional adult symptom networks, interpretation of these is limited, as depression symptoms evolve and fluctuate in severity and interconnectedness over time, forming a dynamic temporal network^1,3^. Intraindividual networks are particularly important to examine across adolescence, where depression onset is most severe and symptoms follow diverse trajectories, but intervention may be most fruitful^4–7^.

In both cross-sectional and longitudinal networks, network evaluation is focused on centrality estimates—measures of node importance in the network^8^. Commonly reported estimates are node strength, closeness and betweenness. However, centrality estimates were originally developed for social network analysis and have more limited interpretability within psychological networks due to issues such as node indistinctiveness (symptom overlap) and the lack of consideration for symptom severity^9^.

Network studies also allude to individual symptoms as treatment targets^5,10–13^. However, targeting a specific node (symptom) may not have the desired effect, potentially failing to address system complexity. For example, a symptom might appear central and be targeted for intervention, but if it is actually a common effect of other symptoms, intervening on it may not disrupt the overall network, as it does not influence other nodes^14^. Effective intervention must also consider timing, hence the importance of longitudinal investigation. Consequently, estimating node centrality becomes less relevant longitudinally as symptoms fluctuate. Instead, it is suggested that we disregard centrality estimates and refocus on global measures that observe the network as a complex system, such as network structure comparison and global connectivity^2,9,15,16^. As such, while centrality measures may be useful for questions based on symptom-specific insights, such as identifying the most strongly connected symptoms, further examination of network-wide characteristics to better understand depression as a system is needed.

According to symptom network theory, more densely connected networks tend to be associated with poorer outcomes^1,17^. Persistent depression is thought to arise from a feedback loop of reinforcing connected symptoms. Supporting this, more densely connected networks were observed in individuals with persistent major depression versus those who remitted^12^. Contrastingly, however, major depression treatment responders showed a larger increase in connectivity after treatment than nonresponders for both pharmacological^18^ and therapeutic interventions^19^. Increased network connectivity may therefore not necessarily be ‘bad’ or pathological but tell us about overall system dynamics such as stability and variability^20^. Just as adverse symptoms can propagate and negatively reinforce a tightly connected network, ‘good’ resilience could also propagate in a positively reinforcing network^19,21^.

A potentially more valuable metric is network temperature^22^. Network temperature is analogous to physical temperature in the solid → liquid → gas energetic state transition described by statistical mechanics, which can be modeled using the Ising model^23,24^. In the Ising model, temperature refers to the level of randomness in the possible states (for example, on or off, 1 or −1 and present or absent) that nodes can take^25^. In depression networks, this randomness can be interpreted as symptom fluctuation. Entropy measures the disorder or uncertainty in the system: high entropy in depression networks indicates a wider and more unpredictable variation in symptoms, whereas low entropy suggests more consistent and predictable symptoms. Therefore, temperature influences global entropy and can provide insights into network or depression stability^22^. When considering a network as an interdependent energetic system, temperature tells us about the energetic organization.

**Fig. 1:**
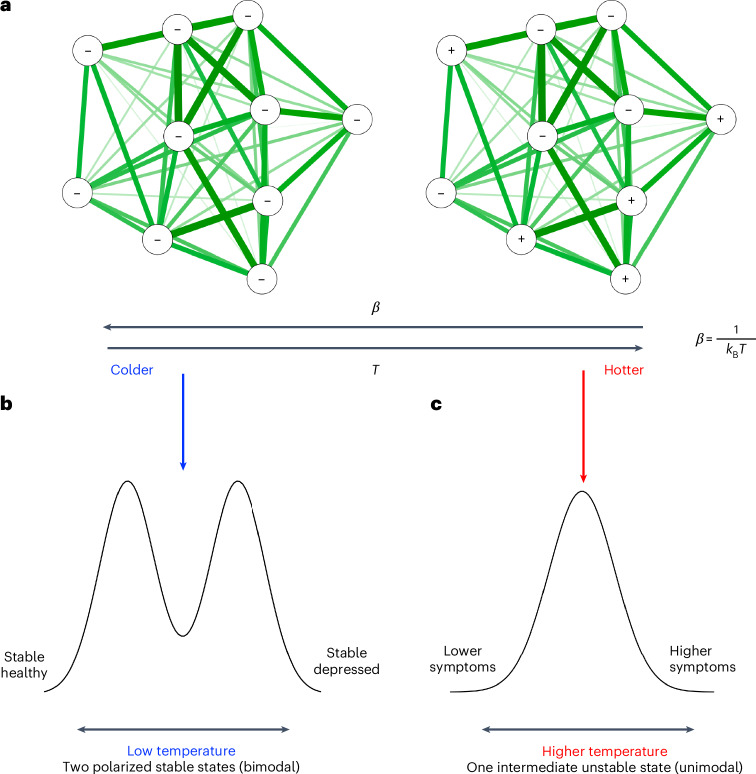
The temperature parameter controlling network behavior in depression. **a**, A lower temperature (higher *β*) characterizes more stable networks with a greater alignment of node states, leading to less variability in symptom configurations. **b**, Low-temperature networks occupy two stable states: one corresponding to a healthy (nondepressed) state and the other to a high-depression state, resulting in a bimodal distribution. **c**, High-temperature networks exhibit greater within-person variability, existing in a single, intermediate unstable state with more fluctuation in symptom configurations.

At low network temperatures, the node states are aligned with a low number of possible configurations (within-person variability), resulting in low entropy and stable, predictable symptom patterns in depression networks. Conversely, high network temperatures lead to more random activation patterns, higher entropy and less stable symptom profiles. In this case, symptoms might vary unpredictably over time, reflecting a system susceptible to change (Fig. 1a).

Network temperature has emerged in causal attitude networks (for example, political attitudes where nodes represent thoughts and feelings)^26–28^ and cognitive belief networks^29^ that apply the Ising model representation of nodes in binary states^24^. These systems tend toward an overall low-energy state of node alignment, where the probability of the overall network state depends on the amount of energy within the network. In attitude networks, directed attention (lower temperature) results in lower attitudinal entropy and increased stability^27^. For example, when a political voter directs their cognitive attention on a specific issue, such as healthcare reform, the variability (entropy) of their attitude toward this issue decreases, leading to a more stable and consistent stance. In belief networks, individuals with high dissonance (belief inconsistency) are associated with low-temperature networks, where tightly connected beliefs amplify the drive to reduce dissonance by changing specific beliefs. Conversely, high temperature networks are characterized by a more malleable belief system but less pressure to resolve inconsistencies^29^. Consequently, temperature represents a global parameter of the network state, scaling both the connectedness of the network, which indicates a general predisposition to an attitude or belief and the thresholds of the network, reflecting external influences on thoughts.

We can bring this idea to psychological symptom networks. The concepts of network temperature and entropy may be valuable in better describing stability in depression and enhancing understanding of risk and resilience. The model of psychiatric disorders as dynamical systems parallels this theory by describing two alternative ‘basins of attraction’ characterized by a resilient healthy and resilient disorder state in early and recent work^15,21,30^. The theory would suggest that both someone who is persistently depressed and someone who never experiences depressive symptoms have low temperature, stable networks in more fixed states (Fig. 1b). Conversely, someone with consistently fluctuating depression state or symptoms would have a high temperature network which exhibits increased variability (Fig. 1c).

We explore the hypothesis that symptom networks stabilize across adolescence. Research shows that emotional regulation improves with age^31^, while emotional fluctuations are pronounced during puberty and adolescence^32^. Depression trajectories also reveal increased symptom heterogeneity during adolescence, but as individuals approach adulthood, they tend to diverge toward two groups: nondepressed and persistently depressed^4,7,33^, suggesting that individual symptoms stabilize over time.

The purpose of this work is to gain insight into the underlying dynamics of adolescent depression by integrating network analysis with longitudinal data. For the first time, we formalize the concept of network temperature in psychological symptom networks and assess its utility for network comparison using a novel approach of multigroup Ising models. We examine how network temperature changes across the development of adolescent depression in three large, independent cohorts, providing a robust understanding of temporal changes. This approach may identify critical developmental periods for intervention. In addition, as a further illustration of this method, we investigate how temperature changes under sex stratification. Sex is a key predictor of depression, with females suffering higher rates and severity across development^34^. This demonstrates the potential application of network temperature to different subgroups or risk factors.

## Results

### Sample characteristics

The imputed sample sizes for each cohort were *N* = 11,726 in the Adolescent Brain and Cognitive Development Study (ABCD), *N* = 9,217 in the Avon Longitudinal Study of Parents and Children Study (ALSPAC) and *N* = 14,958 in the Millennium Cohort Study (MCS) for individuals with at least one depression measure. ABCD is an ethnically diverse cohort and observed self-reported ethnicity of 250 Asian, 1,743 Black, 2,364 Hispanic, 6,134 White and 1,234 other individuals. ALSPAC and MCS are White European cohorts.

Preimputation sample demographics, attrition information for each cohort at each wave and complete case statistics are reported in Table 1. Both the depression summary score means and standard deviations increase with age in the original missing data and complete case samples, indicating an increase in between-person depression score variability. Preimputation symptom frequencies are provided in Supplementary Tables 8–10. Missing symptom data rates were 28.8% in ABCD, 40.1% in ALSPAC and 32.6% in MCS. For ABCD and ALSPAC, the missingness patterns indicated unit nonresponse, whereas in MCS, there was a general pattern of missingness. Full information on missing data patterns is shown in Supplementary Figs. 1–3.

**Table 1:** Preimputed and complete case sample characteristics

		Wave							
	Demographics	1	2	3	4	5	6	7	8
ABCD	*N*	11,232	10,568	10,977	10,510	9,952	9,985	8,218	4,648
	Mean age (years)	10.4	10.9	11.4	12	12.4	12.9	13.4	14.1
	Female (%)	47.68	47.75	47.66	47.63	47.84	47.37	47.59	47.56
	BPM, mean	1.82	1.74	1.54	1.81	1.83	2.02	2.2	2.27
	BPM, s.d.	2.09	2.11	1.94	2.23	2.29	2.38	2.6	2.56
	Complete cases, mean	1.58	1.56	1.32	1.64	1.69	1.9	2.01	2.2
	Complete cases, s.d.	1.89	1.97	1.77	2.08	2.19	2.27	2.47	2.54
ALSPAC	*N*	7,364	6,716	6,019	4,997	4,497	3,335		
	Mean age (years)	10.7	12.8	13.8	16.7	17.8	18.7		
	Female (%)	50.56	51.2	51.52	58.82	56.94	63.83		
	SMFQ, mean	4.04	3.97	4.92	5.91	6.59	6.83		
	SMFQ, s.d.	3.51	3.86	4.49	5.64	5.25	5.93		
	Complete cases, mean	3.77	3.96	4.85	5.53	6.11	6.33		
	Complete cases, s.d.	3.24	3.74	4.4	5.16	4.9	5.58		
MCS	*N* (complete)	10,680	9,378	7,139					
	Mean age (years)	11.2	13.8	16.7					
	Female (%)	51.39	50.85	51.97					
	SDQ Emotional, mean	1.88	2.04	2.03					
	SDQ, s.d.	2	2.13	2.23					
	Complete cases, mean	1.82	1.99	2.03					
	Complete cases, s.d.	1.98	2.11	2.24					

*N*, number of individuals at each wave; s.d., standard deviation.

### Network structure

**Fig. 2:**
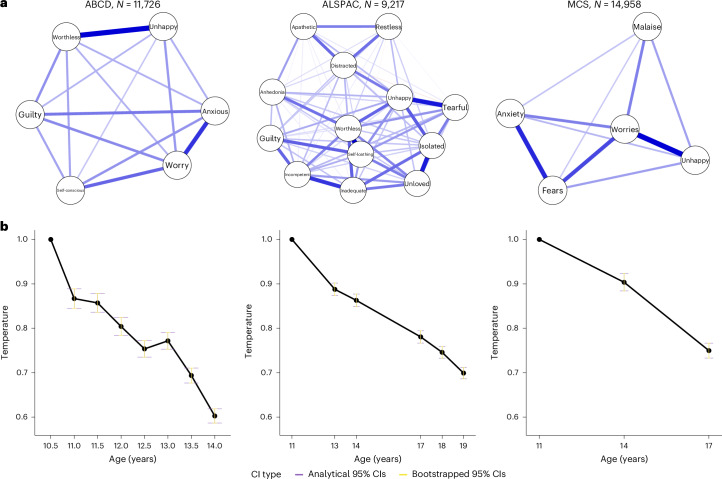
Depression symptom network temperature change across adolescence in three cohorts. **a**, The estimated Ising model networks with equal edges constrained over time. ABCD: *N* = 11,726; ALPSAC: *N* = 9,217; MCS: *N* = 14,958. **b**, The network temperature values were derived from the Ising model at each age, with the first occasion in each cohort fixed to 1 for identification. The error bars represent the 95% CIs around the network temperature parameter estimate, with purple indicating the analytical CIs and yellow indicating the bootstrapped CIs.

Networks were estimated at multiple waves across adolescent development and constructed from Ising models of binary symptoms. For all cohorts, a dense network structure with fixed edge weights that allowed external fields and temperature to vary over time provided the best model fit. The network structures are depicted in Fig. 2a. The full model fit results for models increasing in constraint are reported in Supplementary Tables 11–13. The network model covariance matrices are provided in Supplementary Tables 14–16.

### Network temperature

The results are reported as *T* for temperature with the 95% confidence intervals (CI). Across all three cohorts, the network temperature decreased across adolescence, albeit at different rates (Fig. 2b). Temperature decrease indicates an increase in node state alignment in the network as the system becomes more ordered (less random), and the symptoms tend to ‘all on’ or ‘all off’. In ABCD, the sharpest drop in temperature was between 10.5 (*T* = 1) and 11 years (*T* = 0.87 (0.85–0.88)), with continued cooling to age 14 years (*T* = 0.60 (0.59–0.61)). In ALSPAC, we observed the sharpest drop in temperature between 11 (*T* = 1) and 13 years (*T* = 0.89 (0.88–0.90)) and a consistent cooling between 13 and 19 years (*T* = 0.70 (0.69–0.71)). In MCS, we saw a consistent cooling rate between age 11 (*T* = 1) and 17 years (*T* = 0.75 (0.74–0.76)).

Network entropy decreased consistently with temperature cooling across cohorts, reflecting reduced instability through increased node state alignment (Supplementary Fig. 4). The mean symptom scores and symptom variance showed distinct patterns (increasing in ALSPAC but decreasing in ABCD and MCS), indicating that network-level changes (temperature and entropy) provide insights beyond static symptom measures (Supplementary Tables 17–19).

### Sex differences in depression temperature across adolescence

**Fig. 3:**
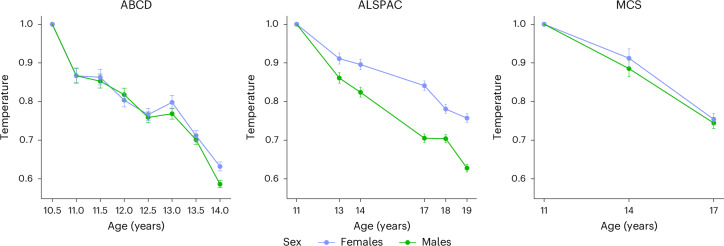
Sex-stratified depression network temperature change across three cohorts. The error bar plots show the relative rate of temperature decrease with age, with the first occasion in each cohort fixed to 1 for identification. ABCD: *N* = 5,603 (females), *N* = 6,120 (males); ALSPAC: *N* = 4,784 (females), *N* = 4,417 (males); MCS: *N* = 6,382 (females), *N* = 6,442 (males). The error bars represent the analytical 95% CIs around the network temperature parameter estimate. The lines illustrate relative rates of cooling across age, not absolute temperature values.

The sex stratification of network temperature revealed an overall trend of faster cooling and lower temperature in males than females across adolescence in all three cohorts (Fig. 3). Female temperature was higher across adolescence, indicating increased symptom variability and, therefore, reduced network stability. This means females observed a longer period of high temperature where the network is more susceptible to change, for example, potential impact by environmental stressors acting as the thresholds.

In the ABCD cohort, temperature decreased at a similar rate for both sexes until mid-adolescence. By age 12.5 years, males exhibited a slightly faster cooling trend compared with females. At this age, the temperatures showed a near identical decrease (males, *T* = 0.76 (0.75–0.77); females, *T* = 0.77 (0.75–0.78)). By age 13 years, the females showed a minor increase in temperature to *T* = 0.80 (0.78–0.82), while the temperature of the males remained stable. By age 14 years, both sexes demonstrated rapid cooling. The males exhibited the greatest cooling rate, with a decrease of *T* = 0.59 (0.58–0.60) compared with the females (*T* = 0.63 (0.62–0.64)). The effect size of the interaction between age and sex was −0.0108, indicating a trend toward faster cooling in males. The evidence for this effect was modest (*P* = 0.050).

In the ALSPAC cohort, the males displayed a rapid cooling rate from age 11 (*T* = 1) to age 14 years (*T* = 0.82 (0.81–0.84)), continuing into late adolescence (*T* = 0.63 (0.62–0.64) at age 19 years). In contrast, the females showed a slower rate of cooling, with temperatures decreasing to *T* = 0.90 (0.88–0.91) by age 14 years. By age 19 years, the relative temperature for the females remained higher compared with the males, at *T* = 0.79 (0.75–0.77), exhibiting less stability in their networks. The effect size of the interaction between age and sex was −0.0140, indicating a faster cooling trend in males, with the evidence supporting this observation being robust (*P* = 0.025).

In the MCS cohort, both the male and female networks exhibited cooling, though males at a slightly faster rate. The difference was most apparent between ages 11 and 14 years, with males cooling to *T* = 0.88 (0.86–0.91) and females to *T* = 0.92 (0.89–0.94). By age 17 years, both sexes reached similar temperatures (males, *T* = 0.75 (0.83–0.76); females, *T* = 0.75 (0.74–0.77)). The effect size for the interaction between age and sex was −0.00162, suggesting a trend toward faster cooling in males. The evidence for this effect was weak (*P* = 0.769).

### Sensitivity analyses

Estimated network structures remained consistent when analyzing nonimputed data (complete cases) only, with the maximum-likelihood estimator (Supplementary Tables 20–22 and Supplementary Figs. 5–7). The network temperature also decreased across all cohorts, albeit to a lesser extent than with the imputed samples. As expected, the network structure remained equivalent with [0,1] encoding. Surprisingly, we also saw consistent results for the temperature parameter suggesting either encoding approach is suitable for analysis of network temperature (Supplementary Tables 23–25 and Supplementary Figs. 8–10). The global network connectivity was approximately proportional to the product of connectivity at the first time point and beta (Supplementary Tables 26–28). The minor discrepancies of calculated values probably reflect numerical variations arising from parameter estimation, aggregation or rounding effects. The overall patterns demonstrate that connectivity is scaled by beta, reinforcing that temperature captures distinct information on system dynamics beyond what connectivity alone can reveal.

## Discussion

For the first time, we apply the concept of network temperature to psychological symptom networks. Specifically, we observed a decrease in the temperature of depressive symptom networks across adolescent development, indicating increased stability and decreased variability of symptoms. While earlier studies have begun to interpret mental health networks as complex systems, moving away from node-focused evaluations, these studies typically identify cross-sectional differences such as network structure or density. We demonstrate network temperature as a novel metric that captures network dynamics over time, a measure currently missing from symptom network studies and which can be applied to group comparisons of network stability.

In three adolescent cohorts with three different depression scales, we show a consistent pattern of network temperature decrease across adolescence. This ‘cooling’ indicates increased within-person depression stability as symptoms become more aligned and less variable with age. This finding is supported by previous work that found global connectivity of depression–anxiety networks increased from childhood through adolescence^35^.

Our results confirm that network temperature and entropy decrease together, supporting the hypothesis of reduced instability as networks settle into fewer configurations. Importantly, changes in symptom variance were cohort specific, highlighting that network temperature provides unique insights into system dynamics not captured by symptom variability alone.

To illustrate these cohort-specific patterns, we observed that symptom variation decreased in ABCD and MCS, suggesting increasing synchronization of symptoms across individuals and more homogeneous samples. In contrast, the increase in symptom variance in ALSPAC indicates greater heterogeneity in symptom dynamics, potentially reflecting a polarization into two subgroups: individuals with persistently high symptoms and those stabilizing at low levels. This aligns with the theory of low-temperature network states, where symptoms converge into ‘all-on’ or ‘all-off’ configurations, consistent with prior trajectory findings in this cohort^7,33,36^.

Temperature may explain why we see strongly connected networks in both depression patients and healthy individuals—offering a dynamical explanation for the conflicting evidence as to whether connectivity is ‘good’ or ‘bad’. Rather than being pathological in symptom networks, as previously thought^15^, increased connectivity may instead represent lower temperature and increased homogeny of symptom states within a group.

In the study suggesting connectivity as pathological, the individuals with persistent depression had a higher network connectivity compared with those whose depression had remitted^12^. This difference may be due to persisters having stable low-temperature networks, while remitters exhibit higher temperatures and more symptom heterogeneity.

In the study that saw increased connectivity as a marker of improvement, major depression treatment responders had a weaker baseline network connectivity than nonresponders but then saw the greatest increase in connectivity with treatment^19^. This could be understood as treatment responders initially having less stable, ‘hotter’ networks, which were more susceptible to intervention. This increased system randomness may have enabled treatment to tip them into a positive stable state, heightening stability or connectivity, whereas the nonresponders began with lower temperature networks and remained ‘stuck’ in these negative low-temperature states.

Connectivity and temperature are conceptually distinct measures in network dynamics. Connectivity quantifies the aggregate strength of pairwise interactions in a static network, while temperature governs the system’s stability and susceptibility to fluctuations, reflecting its dynamism and likelihood of transitioning between configurations. By influencing edge weights and thresholds (external fields), temperature shapes the probability distribution of node states, making it especially relevant in psychological networks where symptom instability is critical.

While connectivity captures the overall strength of relationships, temperature highlights fluctuations and transitions in symptom patterns, offering a nuanced perspective on system dynamics. This distinction is particularly valuable when sudden changes or instability are key phenomena. By identifying temperature and enabling group comparisons, our study complements traditional connectivity measures and provides new insights into psychological network behavior.

Our findings are also consistent with research on longitudinal depression trajectories. Across adolescence, studies observe two stable groups: one displaying persistent symptoms (stable high) and a second with low or no symptoms (stable low)^7,33,37^. Individuals in both of these stable groups would be characterized by low-temperature networks. The individuals belonging to more intermediate trajectories (increasing and decreasing) who experience fluctuating symptom patterns eventually converge into one of these stable high- or stable low-trajectory groups by early adulthood. This aligns with the theory of statistical thermodynamics for complex systems tending to lower-energy states. As adolescents age, within-individual networks homogenize, and the symptom states align.

Conversely, between-individual networks exhibited increased variation across development, as individuals tend toward either pole of the depression spectrum. We observed this polarization as the increased variance in depression summary scores. This supports the theory of depression as a bistable system with two attractor states^15,21^. The novelty that we propose is that these attractor states are both characterized by low-energy states but at opposite poles of the depression spectrum (symptoms ‘all on’ or ‘all off’), as depicted in Fig. 1.

Importantly, both the pattern of network temperature cooling and increased polarization drive home the cruciality of early intervention for mitigating depression onset. The sharp changes in temperature between early to mid-adolescence indicate that when the system is at higher temperatures and displays the most randomness, we are presented with a critical period for intervention before network states become ‘stuck’. Etiologically, this period corresponds to the onset of puberty and hormone changes, synaptic pruning of neural connections and social changes, such as the transition to secondary education^38^.

While our study emphasizes network dynamics over structure, we observed consistent network structure across all cohorts and time points. However, since our model tests are frequentist, this consistency suggests a lack of evidence for differences rather than definitive equality. Nonetheless, the large sample sizes bolster the evidence for truly equal network structures. We found the observed data fit best models with constrained edges but allowing thresholds and temperature to vary with time. This consistent equality was also found in a longitudinal network of depressed mothers^39^. The equal network structure means centrality estimates are also equal over time. The consistency of centrality across development, as indicated by stable network structures over time, supports the transition from a node-focused approach to overall properties of the network, which offers further insight for psychological network analysis.

We also observed sex differences in the temperature of depression networks across adolescence. The males generally exhibited faster cooling and lower temperatures across adolescence compared with the females, though this sex difference was not significant in all cohorts. This pattern suggests that the symptom networks of the males stabilize earlier, while the symptom networks of the females remain more variable and unstable, potentially contributing to higher rates of depression observed in adolescent females^34^. Our findings are supported by longitudinal work in depressive symptoms that show females follow more heterogeneous trajectory patterns with higher peaks across adolescence^40^. Clinically, this may highlight an even stronger need to intervene early in males and continuously in females.

Sex is one predictor of mental health trait differences, but there are many other risk and protective factors that could make use of this analytical approach in either a cross-sectional two-group approach or longitudinally. For example, how does temperature differ in someone with poor sleep versus good sleep? How does temperature vary with substance use? How does temperature respond to depression interventions? These are opportunities for future work to explore both for depression and other psychological conditions.

Our study emphasizes the unique contributions of network models to understanding psychological systems. Unlike traditional approaches such as structural equation modeling, which infer latent constructs from aggregated questionnaire responses, network models focus on dynamic interrelations between individual symptoms, making them useful for exploring the structure and behavior of psychological systems.

While structural equation modeling relies on acyclic (no loops), theory-driven path models^41^, network approaches such as the Ising model can capture potentially cyclic and data-driven dynamics, offering new insights such as network temperature to enrich our understanding of system behavior.

This study had a few limitations. As expected with longitudinal research, there were many missing symptom observations (Table 1). Though our approach to handling missing data using multiple imputation and pooling mode symptom combinations is appropriate for the nature of the data, we cannot guarantee data are missing at random. For example, it is known that those with severe depression are less likely to complete longitudinal follow-up, thus leading to selective attrition bias in the imputed data^42^. Imputation for network analysis data, particularly longitudinal, is still a gap in the methodology literature^43,44^. However, our sensitivity analyses with complete cases only found consistent results for both the model selection and pattern of temperature decrease, albeit less steep, across cohorts (Supplementary Tables 20–22 and Supplementary Figs. 5–7). Though these approaches all have their own biases, the consistent general pattern of results provides robustness.

We leveraged three adolescent cohorts with different symptom scales (Short Mood and Feelings Questionnaire (SMFQ), Strength and Difficulties Questionnaire (SDQ) and Brief Problem Monitoring Youth Scale (BPM)), which had a mix of self-report (ABCD and ALSPAC) and parent report (MCS) and included individuals born in different generations and from different countries. Despite this heterogeneity, we show replicated results of decreasing temperature and sex differences in depression networks strengthening the generalizability of our findings. The diversity in the ABCD cohort allows for broader generalizability across different ethnic groups.

## Conclusions

Overall, our work provides more evidence for the emerging dynamical systems perspective of psychological disorders. We demonstrate network temperature as a novel parameter for assessing the stability of symptom networks over time and reveal early adolescence as a critical window for intervention. Sex differences exemplify how temperature may provide more specificity of subgroup-based dynamics and prevention approaches.

## Methods

### Sample and depression phenotype

The participants were from the ABCD^45,46^ study from the USA, the ALSPAC^47,48^ study from the UK and the MCS^49–51^ study from the UK.

We included all individuals with at least one completed validated depression questionnaire. The longitudinal symptom data available for each cohort differed in measure, frequency and age range. In ABCD, the self-report depression symptoms were acquired from the internalizing subscale of the self-report BPM (six items) collected at eight waves between the ages of 10 and 14 years. In ALSPAC, we used the SMFQ (13 items) collected at six waves between the ages of 11 and 19 years. In MCS, we used the emotional problems subscale from the parent-report SDQ (five items) collected at three waves between the ages of 11 and 17 years. Full information on cohorts, questionnaires and validation of methods can be found in Supplementary Information and Supplementary Tables 1–6.

### Symptom cleaning and multiple imputation

The symptom data for all three cohorts was scored on a three-point Likert scale (not true, sometimes and true). However, due to the Ising model specification, we binarize symptoms to [−1, 1] encoding with −1 meaning ‘not true’ and 1 meaning ‘true’, where ‘sometimes’ is subsumed into ‘true’. Using the MICE R package, we used multiple imputations by chained equations with predictive mean matching to impute missing symptom data with *m* imputed datasets, where *m* is the percentage of missing data and 20 iterations. The imputed datasets were aggregated and pooled point estimates of the mode symptom combination were selected for the final imputed dataset. To determine the impact of both attrition and imputation, we estimated the networks and extracted temperature change for all cohorts with nonimputed (complete cases) datasets using the maximum-likelihood estimator.

### The Ising model

The Ising model provides a framework to understand the probability distribution of different configurations in a network based on the interactions between nodes (symptoms) and external (environmental) influences. It captures how aligned node states (lower energy) are more probable and how temperature affects the randomness in the system. The Ising model equation is given by equation (1) (ref. ^25^)

1 $$P\left({X}={x}\right)=\,\frac{{\mathrm{e}}^{-\beta H({x})}}{Z},$$

where $P\left({X}={x}\right)$ is the probability of the network nodes being in a specific configuration. A configuration *x* represents a specific state of all nodes in the network, where each node *x*_*i*_ can be in one of two states (for example, −1 or 1). *H*(*x*) is the potential function, which measures the energy of the configuration *x* with lower-energy configurations more likely to occur. The potential function captures the effect of external fields on nodes and the interactions between connected nodes. *β* is the inverse temperature parameter that scales the influence of *H*(*x*) on the probability. A high *β* (low temperature) means that the system favors lower-energy states and less randomness. *Z* is the partition function, which sums over all possible network configurations to ensure probabilities sum to 1, acting as a normalization constant.

### Network temperature

Network formulation requires all parameters—edge weights, thresholds and temperature—to be estimated in a series of cross-sectional networks across time. In the first group (time point), *β* (inverse temperature) is unidentified, so we fix it to 1—analogous to the latent variable identification in structural equation modeling^52^. Adding equality constraints to the groups (here, the model at different time points) means that temperature becomes identified from the second group onwards (*t*_2_:*t*_*n*_). Consequently, temperature can increase or decrease relative to this starting value. To determine network temperature change, we extracted the thermodynamic *β* value from the best-fitting Ising model output at each time point. Thermodynamic *β* is the inverse of the temperature of the network represented in the Ising model given by equation (2) (ref. ^53^)

2 $$\beta =\frac{1}{{k}_{\mathrm{B}}T},$$

where *T* is the temperature and *k*_B_ is the Boltzmann constant^53,54^. *β* is also termed the ‘dependence parameter’, as it scales the configuration and, thus, entropy of the Ising model^27,54^. As *β* increases, the probability of the network configuration depends more on its energy, thus resulting in a higher configuration stability and lower within-system variability. The system tends toward lower Gibbs entropy due to the reduced likelihood of higher-energy configurations. Conversely, when *β* is at its lowest, all network configurations are equally probable, indicating a maximum Gibbs entropy.

We computed temperature as 1/*β* and plot the change in temperature across adolescence for each cohort. Importantly, temperature and entropy, while related, are distinct: temperature reflects the stability of the system and can fluctuate independently of overall entropy. For example, even if temperature increases (indicating higher instability), it does not necessarily imply an increase in the system’s Gibbs entropy if the overall distribution of configurations remains relatively stable.

We performed 1,000 bootstraps of network temperature estimates to derive bootstrapped 95% CIs in addition to analytical CIs.

### Network estimation and model selection

Depression networks were estimated using multigroup Ising models of binary symptoms. We used the Ising model function in the psychonetrics R package to estimate network models independently at different time points. We estimated four network models in each cohort in an iterative approach with increasing constraints: (1) fully saturated with all parameters free; (2) set omega equal between groups for equal edges (structure) over time; (3) set omega and tau equal between groups for equal edges and external information (thresholds) over time, respectively; and (4) set omega, tau and beta equal between groups for equal edges, thresholds and temperature over time, respectively (Supplementary Table 7). For each of these four models, we also tested both a dense network (all nodes connected) and a sparse network (pruning of edges) structure, resulting in eight models estimated for each cohort. This approach has been detailed in previous Ising model methodology^22^. The estimated network structures were plotted with the qgraph R package.

The best model was selected on the basis of fit parameters including low Akaike information criterion, low Bayesian information criterion, root mean square error of approximation <0.05 and *P* value (*α* = 0.05) of the chi-squared test. The root mean square error of approximation compares subsequent models of increasing constraints with the fully connected (saturated) Ising model of pairwise interactions, which has 0 degrees of freedom. This approach is analogous to structural equation modeling. We performed a pairwise nested comparison between dense and sparse networks, where model 1 is the reference model. The model descriptions and nesting structure are detailed in the Supplementary Table 7.

We derived network entropy using the IsingSampler::IsingEntropy R package and function and calculated the mean symptom scores and variance across time to compare observable symptom changes with temperature and entropy.

### Stratification application and sex differences

We computed the temperature change independently in males and females for the three cohorts to provide an example of how network temperature can be investigated in stratified groups. The networks were estimated, and the temperature was extracted as described for the main analysis.

We used a linear mixed-effects regression model with temperature as the outcome, age, sex and their interaction (age × sex) as fixed effects, and a random intercept for age to examine the interaction between age and sex on network temperature. This allowed us to examine how the rate of change in network temperature differs between males and females over time. The *P* values are two-sided with *α* = 0.05.

### Sensitivity analyses

The classical Ising model denotes binary nodes as [−1, 1]; however, network psychometricians often prefer [0, 1] encoding where symptoms are conceptualized as present or absent^15^. Though differently encoded networks can be transformed to produce statistically equivalent models, network parameters generate different dynamical interpretations^55^. Under [−1, 1] encoding, the dependence parameter (*β*) controls the probability of node state alignment. As *β* increases, the probability of nodes aligning as [1, 1] or [−1, −1] increases if these nodes are connected positively in the network. However, under [0, 1] encoding, only the probability of [1, 1] increases with *β*, not [0, 0]. We tested the impact of [0, 1] encoding on temperature to determine whether this parameter requires specific encoding. We followed advised reporting methods for psychological network analysis where applicable^56^.

We used the NetworkComparisonTest R package to derive global network connectivity and compare it with temperature across time points to further assess the validity and novelty of temperature. Since the networks in our analysis are structurally identical with equal edge weights, we expect connectivity to provide no additional information, with any observed differences driven by beta (inverse temperature). If network structures differed, connectivity could vary independently of temperature, with temperature potentially amplifying or suppressing connectivity effects.

### Reporting summary

Further information on research design is available in the Nature Portfolio Reporting Summary linked to this article.

## Supplementary information

Supplementary Information Supplementary Tables 1–28 and Figs. 1–10.

Reporting Summary

## Data Availability

The ALSPAC study website contains details of all the data that are available through a fully searchable data dictionary at http://www.bristol.ac.uk/alspac/researchers/access/. Permission to use the ALSPAC data is obtained through a proposal system managed by the ALSPAC executive. The ABCD data used in the preparation of this article were obtained from the ABCD study at https://abcdstudy.org, held in the NIMH Data Archive. The ABCD data repository grows and changes over time. The ABCD data used in this report came from the NIMH Data Archive at 10.15154/8873-zj65. The DOIs can be found at https://nda.nih.gov/abcd/. The MCS data are freely available and can be downloaded on the UK Data Service website at https://ukdataservice.ac.uk/.

## Code availability

All code and analysis scripts are available via GitHub at https://github.com/poppyzenzi/network_temperature.
