# Supplementary material for: Network temperature as a metric of stability in depression symptoms across adolescence
Source: Nat Ment Health. 2025 Apr 29;3(5):548–57. doi: 10.1038/s44220-025-00415-5 (PMC12066352; doi:10.1038/s44220-025-00415-5)
Supplement: Supplementary file 1 — Supplementary Tables 1–28 and Figs. 1–10. [file 44220_2025_415_MOESM1_ESM.pdf]

nature mental health

Supplementary information

https://doi.org/10.1038/s44220-025-00415-5

Network temperature as a metric of stability in depression symptoms across adolescence

In the format provided by the authors and unedited

Supplementary Information

Network Temperature as a Metric of Stability in Depression Symptoms Across Adolescence

1. Sample and symptom descriptives

ABCD and the Brief Problem Monitoring (Youth) scale

The Adolescent Brain and Cognitive Development (ABCD)[1,2] study cohort has recruited a total number of 11,876 children starting in 2015 across 21 different North American study sites[1]. The study was approved by the National Institute of Mental Health Data Archive, United States (NIMH). Written consent was obtained from all participants. Data was accessed through the NDA database (https://nda.nih.gov/abcd/); Federal-Wide Assurance: FWA00018101). Full descriptives are in **Table S1.**

Table S1 | ABCD depression scores and cohort descriptives for 8 occasions

Time Point	Sample Size	Mean Age	Mean BPM (SD)
1	11232	10.398	1.817 (2.089)
2	10568	10.924	1.742 (2.113)
3	10977	11.396	1.539 (1.944)
4	10510	12.027	1.814 (2.227)
5	9952	12.408	1.825 (2.294)
6	9985	12.913	2.022 (2.377)
7	8218	13.403	2.203 (2.601)
8	4648	14.081	2.270 (2.561)

The Brief Problem Monitoring (internalising) is a 6-symptom subscale with items derived using factor analysis and item response theory from CBCL and YSR [3]. BPM has a clinical T-score cut off of >65 (from the ASEBA manual). Internal consistency and correspondence with CBCL has been demonstrated [4]. The Norwegian translated version of the BPM has been validated for good internal reliability and has convergent validity with both the full ASEABA scale [5] and the Behaviour and Feelings Survey [6]. BPM items are listed in **Table S2.**

Table S2 |Brief Problem Monitoring (Youth)

	BPM items
1	I feel worthless or inferior
2	I am too fearful or anxious
3	I feel too guilty
4	I am self-conscious or easily embarrassed
5	I am unhappy, sad, or depressed
6	I worry a lot

ALSPAC and the Short Mood and Feelings Questionnaire

Please note that the ALSPAC[7,8] study website contains details of all the data that is available through a fully searchable data dictionary and variable search tool (http://www.bristol.ac.uk/alspac/researchers/our-data/). Ethical approval for the study was obtained from the ALSPAC Ethics and Law Committee and the Local Research Ethics Committees. Consent for biological samples has been collected in accordance with the Human Tissue Act (2004). Informed consent for the use of data collected via questionnaires and clinics was obtained from participants following the recommendations of the ALSPAC Ethics and Law Committee at the time. Pregnant women resident in Avon, UK with expected dates of delivery between 1st April 1991 and 31st December 1992 were invited to take part in the study. The initial number of pregnancies enrolled was 14,541 with 13,988 children who were alive at 1 year of age. The total sample size for analyses using any data collected after the age of seven is therefore 15,447 pregnancies, resulting in 15,658 foetuses. Of these 14,901 children were alive at 1 year of age. Full descriptives are in **Table S3.**

Table S3 | ALSPAC depression scores and cohort descriptives for 6 occasions

Time Point	Sample Size	Mean Age	Mean SMFQ (SD)
1	7364	10.648	4.04 (3.51)
2	6716	12.811	3.97 (3.86)
3	6019	13.835	4.92 (4.49)
4	4997	16.679	5.91 (5.64)
5	4997	17.8	6.59 (5.25)
6	3335	18.7	6.83 (5.93)

The Short Mood and Feelings Questionnaire has a clinical cut-off as a total score of >=11. SMFQ has been clinically validated against ICD-10 depression diagnosis [9,10]. SMFQ items are in **Table S4**.

Table S4 | Short Mood and Feelings Questionnaire

	SMFQ items
1	I felt miserable or unhappy
2	I didn’t enjoy anything at all
3	I felt so tired I just sat around and did nothing
4	I was very restless
5	I felt I was no good any more
6	I cried a lot
7	I found it hard to think properly and concentrate
8	I hated myself
9	I was a bad person
10	I felt lonely
11	I thought nobody really loved me
12	I thought I could never be as good as other kids
13	I did everything wrong

MCS and the Strength and Difficulties Questionnaire

The Millennium Cohort Study (MCS)[11,12], which began in 2000 (birth year of participants between 2000-02), is conducted by the Centre for Longitudinal Studies (CLS). It aims to chart the conditions of social, economic and health advantages and disadvantages facing children born at the start of the 21st century The study has been tracking the 'Millennium children' through their early childhood years and plans to follow them into adulthood. It also provides a basis for comparing patterns of development with the preceding cohort studies the National Child Development Study (NCDS) and the 1970 Birth Cohort Study (BCS70). Full descriptives are in **Table S5.**

The MCS was approved by the South West and London Multi-Centre Research Ethics Committees. The Millennium Cohort Study obtained informed written consent from parent/ guardians of the cohort children in order to participate in the study, children themselves as they grow-up and other participants as necessary.

Table S5 | MCS depression scores and cohort descriptives for 3 occasions

Time Point	Sample Size	Mean Age	Mean SDQ (SD)
1	10,680	11.2	3.19 (3.13)
2	9,378	13.8	3.73 (3.40)
3	7,139	16.7	3.77 (4.47)

The Strengths and Difficulties Questionnaire (SDQ) is a brief behavioural screening questionnaire about 2- to 17-year-olds. The emotional symptoms subscale is used to measure information about mood and feelings related to depression. This subscale has a clinical cut-off of >= 5 for depression status[13,14]. SDQ items for the emotional subscale are in **Table S6**.

Table S6 | Strength and Difficulties Questionnaire (emotional subscale)

	SDQ items
1	I get a lot of headaches, stomach-aches, or sickness
2	I worry a lot
3	I am often unhappy, downhearted, or tearful
4	I am nervous in new situations
5	I have many fears, I am easily scared

2. Model description and nesting

Model 1 is the reference. Models 3,5 and 7 are compared with model 1 and models 4,6, and 8 are compared with model 2.

Table S7 | Model descriptions and nesting (grey and white colours represent nested models)

Model	
1	fully saturated model, all parameters free (dense)
2	fully saturated model, all parameters free (sparse)
3	network structure (edge weights) constrained equal (dense)
4	network structure (edge weights) constrained equal (sparse)
5	structure and thresholds (external fields) constrained equal (dense)
6	structure and thresholds (external fields) constrained equal (sparse)
7	structure, thresholds and temperature constrained equal (dense)
8	structure, thresholds and temperature constrained equal (sparse)

3. Symptom endorsement frequency tables (pre-imputation)

Table S8 | ABCD

	Wave							
**Symptom**	**1**	**2**	**3**	**4**	**5**	**6**	**7**	**8**
anxious	3338	2679	2790	2745	2754	3019	2791	1619
guilty	1550	1430	1181	1477	1362	1547	1432	798
self-conscious	4231	4090	4286	4453	4378	4623	4080	2288
unhappy	2000	1545	1301	1485	1455	1761	1717	1016
worry	4337	4329	3860	4409	3935	4355	3578	2170
worthless	1480	1176	960	1142	1193	1272	1389	753

Table S9 | ALSPAC

	Wave					
**Symptom**	**1**	**2**	**3**	**4**	**5**	**6**
anhedonia	1005	1019	1198	936	1155	1027
apathetic	3083	3055	3131	2786	3112	2228
distracted	4065	3193	3441	2773	3291	1950
guilty	788	989	1181	1169	1298	728
inadequate	1933	1414	1614	1365	1415	1073
incompetent	1122	1033	1149	1201	1276	915

isolated	2391	1642	1881	2114	2170	1640
restless	3648	3122	3223	2474	2803	1985
self-loathing	968	871	1079	1158	1090	694
tearful	1303	1086	1176	1411	1421	1019
unhappy	3957	4057	3765	3571	3156	2412
unloved	890	793	1017	1071	1098	577
worthless	1175	985	1273	1306	1362	1058

Table S10 | MCS

	Wave		
**Symptom**	**1**	**2**	**3**
anxiety	4884	4182	3023
fears	3776	2894	2045
malaise	4162	3929	2675
unhappy	2094	2198	1847
worries	4634	4396	3696

4. Missing data patterns

Figure S1 | Missing symptoms and sex observations in the ABCD study

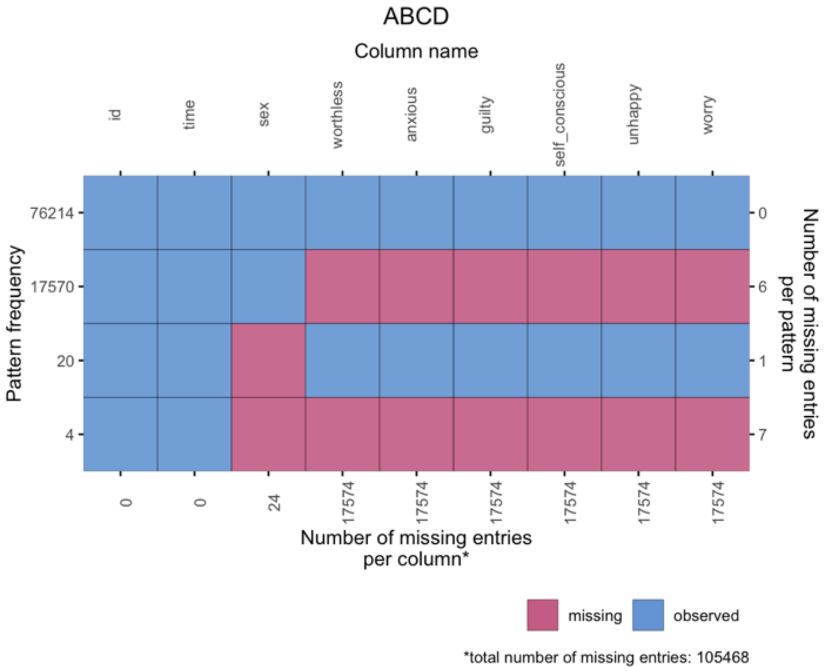

Figure S2 | Missing symptom and sex observations in the ALSPAC study

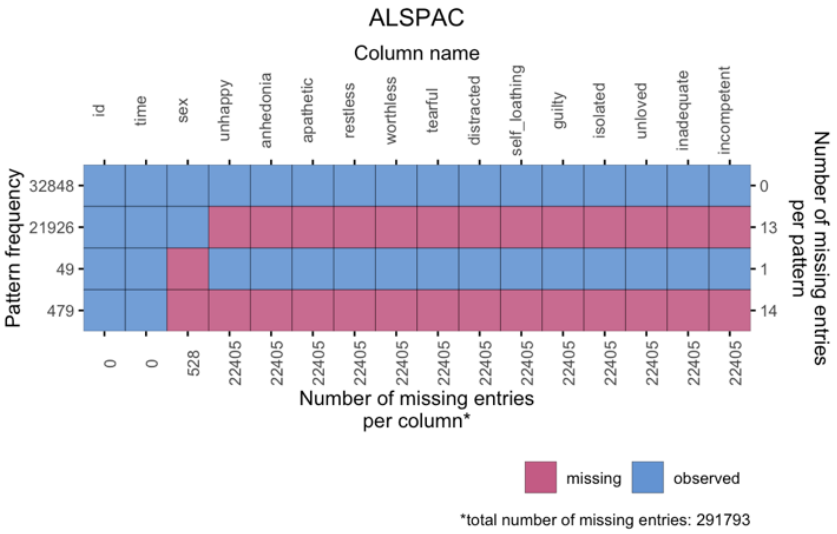

Figure S3 | Missing symptom and sex observations in the MCS study

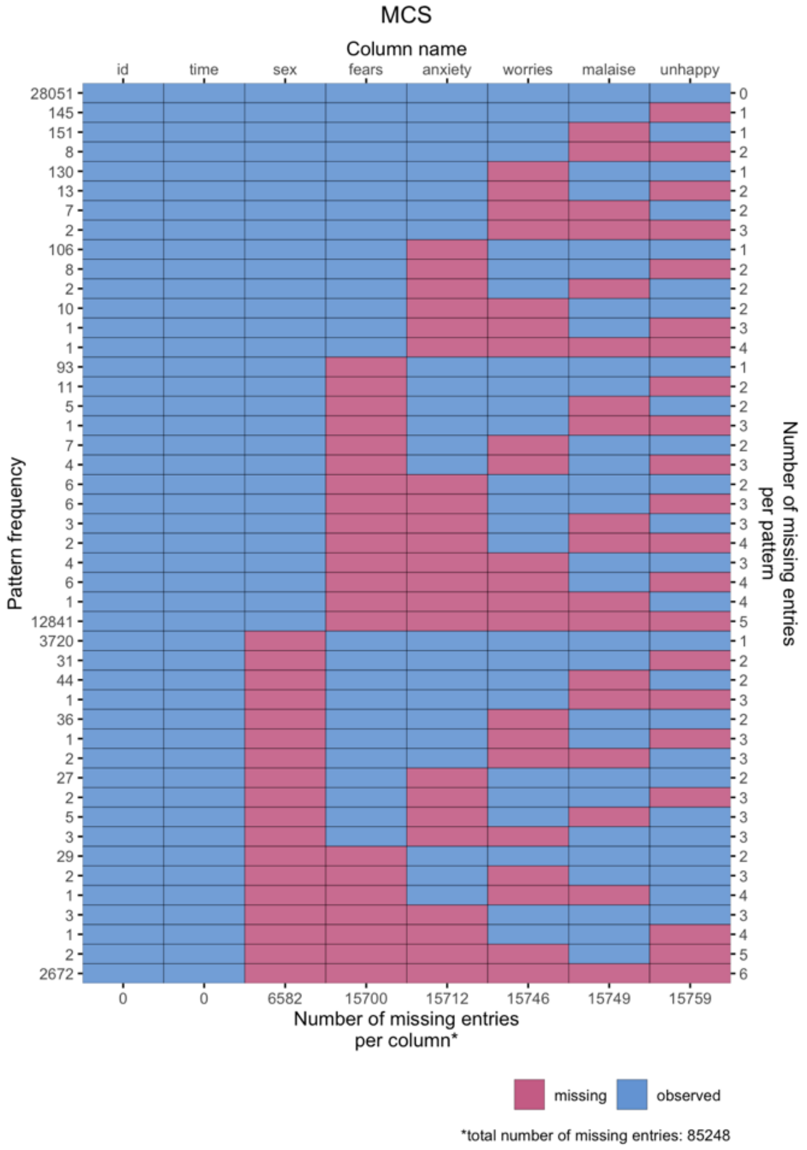

5. Model fit parameters for the main analysis

Table S11 | ABCD

Model	DF	AIC	BIC	RMSEA	Chisq	Chisq_diff	DF_diff	p_value
3. equal networks (dense)	98	445748.11	446409.54	0.015	348.9	348.9	98	<0.0001
4. equal networks (sparse)	98	445748.11	446409.54	0.015	348.9	-	-	-
1. all parameters free (dense)	0	445595.21	447182.64	~	~	~	~	~
2. all parameters free (sparse)	0	445595.21	447182.64	~	~	~	~	~
5. equal networks and thresholds (dense)	140	446994.59	447259.17	0.031	1679.58	1330.48	42	<0.0001
6. equal networks and thresholds (sparse)	140	446994.59	447259.17	0.031	1679.58	-	-	-
7. all parameters equal (dense)	147	449865.94	450064.64	0.051	4564.73	2885.34	5	<0.0001
8. all parameters equal (sparse)	147	449865.94	450064.64	0.051	4564.73	-	-	-

Table S12 | ALSPAC

Model	DF	AIC	BIC	RMSEA	Chisq	Chisq_diff	DF_diff	p_value
4. equal networks (sparse)	394	537848.14	539204.06	0.022	2174.96	9.52	9	0.39
3. equal networks (dense)	385	537856.61	539292.82	0.022	2165.43	2007.94	273	<0.0001
2. all parameters free (sparse)	112	536394.68	540266	0.0066	157.5	157.5	112	0.003
1. all parameters free (dense)	0	536461.18	541331	~	~	~	~	~
6. equal networks and thresholds (sparse)	460	540718.14	541485.35	0.033	5176.96	1368.05	5	<0.0001
5. equal networks and thresholds (dense)	450	540722.61	541268.85	0.031	5588.75	8558.75	56	<0.0001
8. all parameters equal (sparse)	465	542089.93	542812.97	0.038	6545.02	1381.58	5	<0.0001
7. all parameters equal (dense)	455	542096.2	542907	0.038	6545.02	-	-	-

Table S13 | MCS

Model	DF	AIC	BIC	RMSEA	Chisq	Chisq_diff	DF_diff	p_value
3. equal networks (dense)	18	187291.59	187526.8	0.015	76.88	76.88	18	<0.0001
4. equal networks (sparse)	18	187291.59	187526.8	0.015	76.88	-	-	-
1. all parameters free (dense)	0	187250.71	187642.74	~	~	~	~	~
2. all parameters free (sparse)	0	187250.71	187642.74	~	~	~	~	~
5. equal networks and thresholds (dense)	28	187836.87	187894.08	0.031	642.15	565.28	10	<0.0001
6. equal networks and thresholds (sparse)	28	187836.87	187894.08	0.031	642.15	-	-	-
7. all parameters equal (dense)	30	188893.81	189114.48	0.063	1793.1	1150.94	30	<0.0001
8. all parameters equal (sparse)	30	188893.81	189114.48	0.063	1793.1	-	-	-

6. Network model covariance matrices

Table S14 | ABCD network matrix

	worthless	anxious	guilty	self-conscious	unhappy	worry
worthless	0.000	0.133	0.184	0.151	0.424	0.130
anxious	0.133	0.000	0.225	0.179	0.136	0.339
guilty	0.184	0.225	0.000	0.154	0.124	0.197
self-conscious	0.151	0.179	0.154	0.000	0.097	0.257
unhappy	0.424	0.136	0.124	0.097	0.000	0.172
worry	0.130	0.339	0.197	0.257	0.172	0.000

Table S15 | ALSPAC network matrix

	unhappy	anhedonia	apathetic	restless	worthless	tearful	distracted	self_loathing	guilty	isolated	unloved	inadequate	incompetent
unhappy	0.000	0.107	0.099	0.097	0.220	0.329	0.191	0.160	0.120	0.233	0.088	0.082	0.059
anhedonia	0.107	0.000	0.138	0.049	0.203	0.068	0.060	0.050	0.092	0.083	0.082	0.027	0.109
apathetic	0.099	0.138	0.000	0.183	0.027	0.037	0.211	-0.011	0.018	0.051	0.011	0.021	0.029
restless	0.097	0.049	0.183	0.000	0.063	0.015	0.205	-0.014	0.077	0.072	-0.001	0.003	0.029
worthless	0.220	0.203	0.027	0.063	0.000	0.155	-0.012	0.362	0.045	0.084	0.173	0.225	0.172
tearful	0.329	0.068	0.037	0.015	0.155	0.000	-0.033	0.146	0.054	0.185	0.081	-0.001	0.103
distracted	0.191	0.060	0.211	0.205	-0.012	-0.033	0.000	0.061	0.082	0.094	-0.005	0.105	0.094
self_loathing	0.160	0.050	-0.011	-0.014	0.362	0.146	0.061	0.000	0.226	0.088	0.183	0.138	0.117
guilty	0.120	0.092	0.018	0.077	0.045	0.054	0.082	0.226	0.000	0.086	0.069	0.089	0.220
isolated	0.233	0.083	0.051	0.072	0.084	0.185	0.094	0.088	0.086	0.000	0.325	0.208	0.070
unloved	0.088	0.082	0.011	-0.001	0.173	0.081	-0.005	0.183	0.069	0.325	0.000	0.194	0.144
inadequate	0.082	0.027	0.021	0.003	0.225	-0.001	0.105	0.138	0.089	0.208	0.194	0.000	0.288
incompetent	0.059	0.109	0.029	0.029	0.172	0.103	0.094	0.117	0.220	0.070	0.144	0.288	0.000

Table S16| MCS network matrix

	malaise	worries	unhappy	anxiety	fears
malaise	0.000	0.237	0.178	0.114	0.102
worries	0.237	0.000	0.477	0.240	0.344
unhappy	0.178	0.477	0.000	0.136	0.182
anxiety	0.114	0.240	0.136	0.000	0.406
fears	0.102	0.344	0.182	0.406	0.000

7. Network entropy

Figure S4 | Change in network entropy across development in three cohorts. ABCD N=11,726, ALSPAC N=9217, MCS N=14958. X axes represent age in years and Y axes represent calculated network entropy.

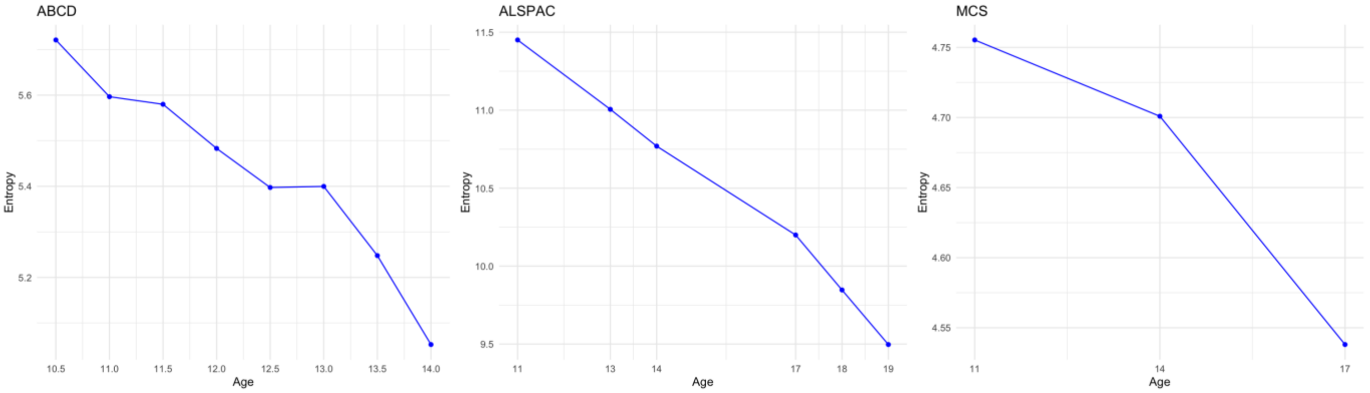

8. Symptom mean and variance

Table S17 | Change in mean symptom scores and symptom variance in ABCD

ABCD		
**Age**	**OverallSymptomMean**	**MeanSymptomVariance**
10.5	-0.518	0.690
11	-0.565	0.633
11.5	-0.590	0.600
12	-0.552	0.640
12.5	-0.566	0.629
13	-0.520	0.673
13.5	-0.560	0.648
14	-0.705	0.477

Table S18| Change in mean symptom scores and symptom variance in ABCD

ALSPAC		
**Age**	**OverallSymptomMean**	**MeanSymptomVariance**
11	-0.532	0.629
13	-0.559	0.594
14	-0.504	0.648
17	-0.469	0.659
18	-0.407	0.685
19	-0.419	0.658

Table S19 | Change in mean symptom scores and symptom variance in ABCD

MCS		
**Age**	**OverallSymptomMean**	**MeanSymptomVariance**
11	-0.482	0.751
14	-0.521	0.715
17	-0.626	0.600

9. Network and temperature estimation with non-imputed data

Listwise deletion was applied in *psychonetrics* to run the models on complete cases only from the original data. This sensitivity uses the default estimator='ML' in the Ising model.

Table S20 | ABCD network estimation with non-imputed data

model	DF	AIC	BIC	RMSEA	Chisq	Chisq_diff	DF_diff	p_value
3. equal networks (dense)	98	410120.44	410767.35	0.013	263.83	263.83	98	< 0.0001
4. equal networks (sparse)	98	410120.44	410767.35	0.013	263.83	~	~	1
1. all parameters free (dense)	0	410052.61	411605.19	~	~	~	~	1
2. all parameters free (sparse)	0	410052.61	411605.19	~	~	~	~	1
5. equal networks and thresholds (dense)	140	411495.8	411754.57	0.034	1723.19	1459.36	42	< 0.0001
6. equal networks and thresholds (sparse)	140	411495.8	411754.57	0.034	1723.19	~	~	1
7. all parameters equal (dense)	147	411872.74	412066.82	0.037	2114.13	390.94	7	< 0.0001
8. all parameters equal (sparse)	147	411872.74	412066.82	0.037	2114.13	~	~	1

Figure S5 | ABCD temperature change with non-imputed data (N=3588). The X axis represents age in years and Y axis represents temperature. Error bars represent 95% confidence intervals.

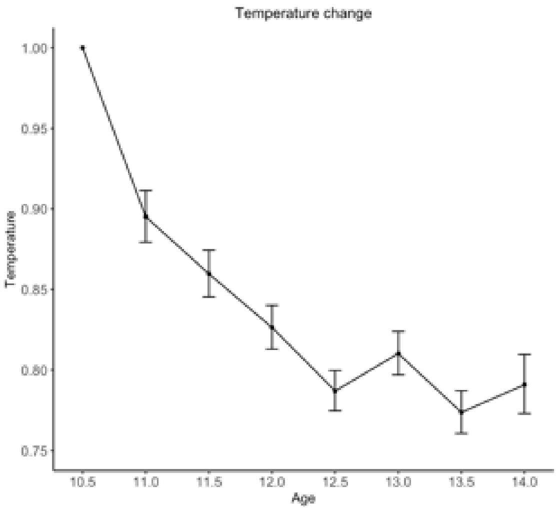

Table S21 | ALSPAC network estimation with non-imputed data

model	DF	AIC	BIC	RMSEA	Chisq	Chisq_diff	DF_diff	p_value
4. equal networks (sparse)	395	386930.25	388198.82	0.026	1810.75	11.44	10	0.32
3. equal networks (dense)	385	386938.81	388291.4	0.026	1799.31	1636.69	262	< 0.0001
2. all parameters free (sparse)	123	385826.12	389379.8	0.0077	162.62	162.62	123	0.0097
1. all parameters free (dense)	0	385909.5	390496.52	~	~	~	~	~
6. equal networks and thresholds (sparse)	461	391079.43	391793.52	0.047	6091.92	146.14	6	< 0.0001
5. equal networks and thresholds (dense)	450	391084.43	391890.94	0.048	6074.93	4264.18	55	< 0.0001
8. all parameters equal (sparse)	466	391232.14	391904.23	0.048	6254.63	162.71	5	< 0.0001
7. all parameters equal (dense)	455	391237.57	392002.07	0.048	6238.07	163.14	5	< 0.0001

Figure S6 | ALSPAC temperature change with non-imputed data (N=1727). The X axis represents age in years and Y axis represents temperature. Error bars represent 95% confidence intervals.

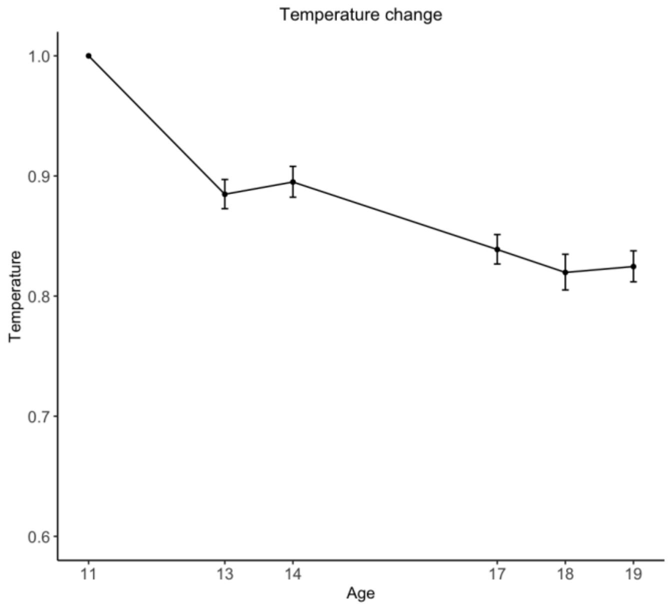

Table S22 | MCS network estimation with non-imputed data. MCS has attrition weights which were not included in our analysis to allow consistent data treatment methods across cohorts.

model	DF	AIC	BIC	RMSEA	Chisq	Chisq_diff	DF_diff	p_value
3. equal networks (dense)	18	172648.23	172874.12	0.0085	31.64	31.64	18	0.024
4. equal networks (sparse)	18	172648.23	172874.12	0.0085	31.64	~	~	1
1. all parameters free (dense)	0	172652.59	173029.08	~	~	~	~	1
2. all parameters free (sparse)	0	172652.59	173029.08	~	~	~	~	1
7. all parameters equal (dense)	30	173207.05	173332.54	0.043	614.45	18.83	2	< 0.0001
8. all parameters equal (sparse)	30	173207.05	173332.54	0.043	614.45	~	~	1
5. equal networks and thresholds (dense)	28	173192.22	173334.44	0.044	595.62	563.98	10	< 0.0001
6. equal networks and thresholds (sparse)	28	173192.22	173334.44	0.044	595.62	~	~	1

Figure S7 | MCS temperature change with non-imputed data (N=5920). The X axis represents age in years and Y axis represents temperature. Error bars represent 95% confidence intervals.

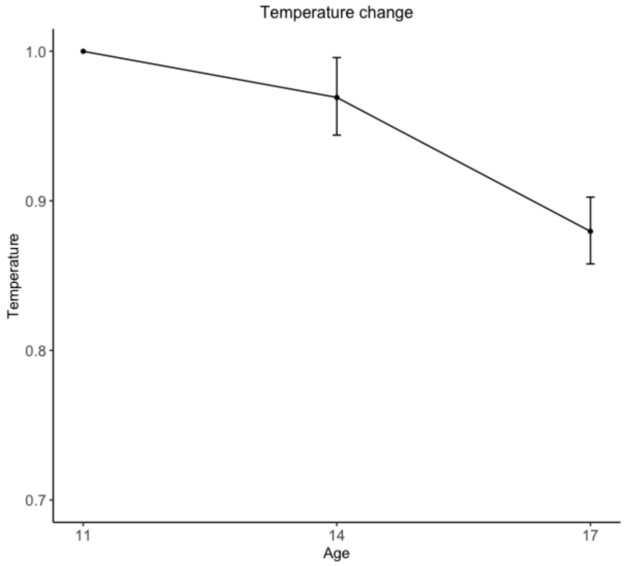

10. Alternative encoding

Results for model and temperature with [0,1] encoding of the Ising model

Table S23 | ABCD network estimation with [0,1] encoding

model	DF	AIC	BIC	RMSEA	Chisq	Chisq_diff	DF_diff	p_value
3. equal networks (dense)	98	445848.11	446509.54	0.013	307.02	307.02	98	< 0.0001
4. equal networks (sparse)	98	445848.11	446509.54	0.013	307.02	~	~	1
1. all parameters free (dense)	0	445737.1	447324.52	~	0	~	0	1
2. all parameters free (sparse)	0	445737.1	447324.52	~	0	~	0	1
5. equal networks and thresholds (dense)	140	447189.52	447454.09	0.031	1732.42	1425.41	42	< 0.0001
6. equal networks and thresholds (sparse)	140	447189.52	447454.09	0.031	1732.42	~	0	1
7. all parameters equal (dense)	147	449747.58	449946.01	0.049	4304.48	2572.06	7	< 0.0001
8. all parameters equal (sparse)	147	449747.58	449946.01	0.049	4304.48	~	0	1

Figure S8 | ABCD temperature change under [0,1] encoding (N=11726). The X axis represents age in years and Y axis represents temperature. Error bars represent 95% confidence intervals.

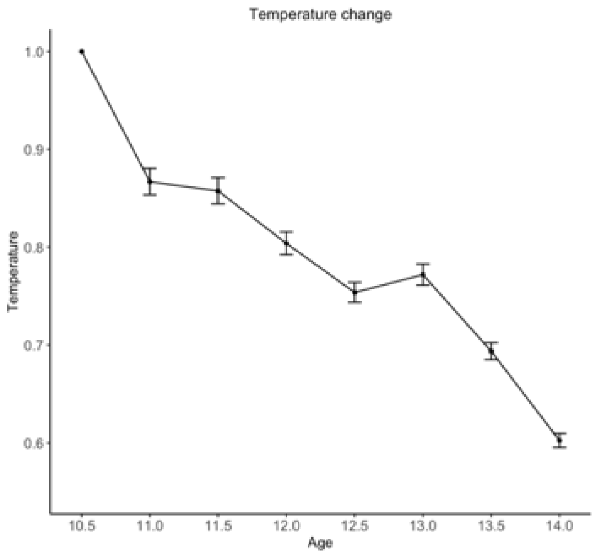

Table S24 | ALSPAC network estimation with [0,1] encoding

model	DF	AIC	BIC	RMSEA	Chisq	Chisq_diff	DF_diff	p_value
4. equal networks (sparse)	393	528995.18	530358.69	0.024	2411.51	6.61	8	0.58
3. equal networks (dense)	385	529004.57	530439.38	0.024	2404.9	2229.26	265	< 0.0001
2. all parameters free (sparse)	120	527305.3	531101.76	0.0071	175.64	175.64	120	0.00071
1. all parameters free (dense)	0	527369.67	532235.54	~	~	~	~	~
6. equal networks and thresholds (sparse)	458	534265.58	535049.83	0.042	7811.92	1140.16	3	< 0.0001
5. equal networks and thresholds (dense)	450	534535.31	535390.84	0.043	8065.64	5654.13	57	< 0.0001
8. all parameters equal (sparse)	464	535404.53	536135.3	0.045	8962.86	1150.94	6	< 0.0001
7. all parameters equal (dense)	455	535411.74	536222.72	0.045	8952.08	886.44	5	< 0.0001

Figure S9 | ALSPAC temperature change under [0,1] encoding (N=9217). The X axis represents age in years and Y axis represents temperature. Error bars represent 95% confidence intervals.

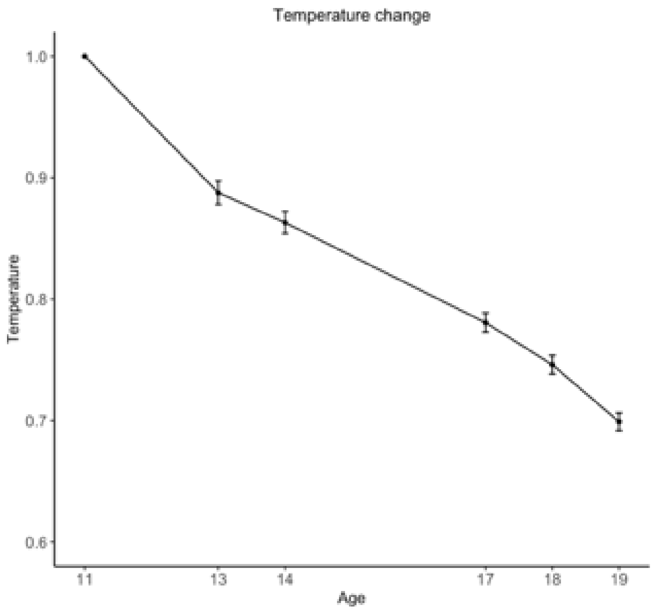

Table S25 | MCS network estimation with [0,1] encoding

model	DF	AIC	BIC	RMSEA	Chisq	Chisq_diff	DF_diff	p_value
3. equal networks (dense)	18	182326.05	182559.5	0.015	71.22	71.22	18	< 0.0001
4. equal networks (sparse)	18	182326.05	182559.5	0.015	71.22	~	0	1
1. all parameters free (dense)	0	182290.83	182679.92	~	~	~	~	~
2. all parameters free (sparse)	0	182290.83	182679.92	~	~	~	0	1
5. equal networks and thresholds (dense)	28	182881.03	183028.02	0.04	646.2	574.98	10	< 0.0001
6. equal networks and thresholds (sparse)	28	182881.03	183028.02	0.04	646.2	~	0	1
7. all parameters equal (dense)	30	183840.01	183969.7	0.061	1609.18	962.98	2	< 0.0001
8. all parameters equal (sparse)	30	183840.01	183969.7	0.061	1609.18	~	0	1

Figure S10 | MCS temperature change under [0,1] encoding (N=14958). The X axis represents age in years and Y axis represents temperature. Error bars represent 95% confidence intervals.

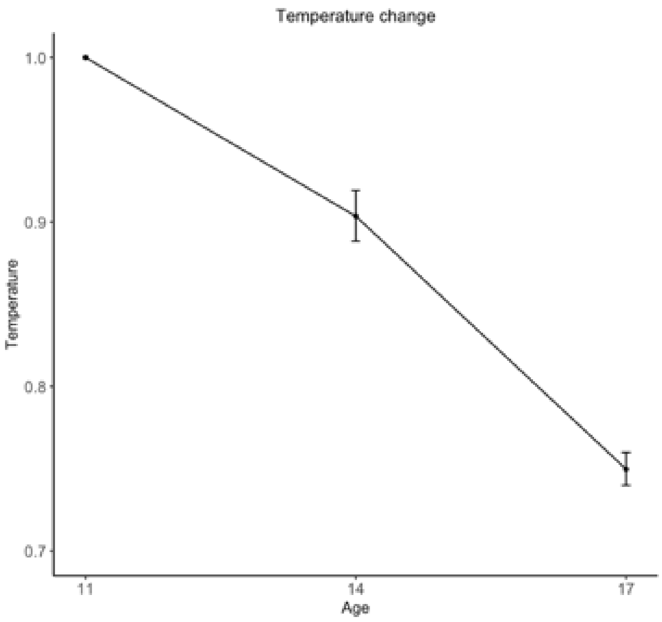

11. Comparison with global connectivity

We derived global network connectivity across time points (**NCT connectivity**) and show it is approximately equal to the product of connectivity at t1 and beta (**connectivity@t1 * beta@tn**). Minor discrepancies likely reflect numerical variations in estimation, rounding or aggregation in network calculations.

Table S26 | Connectivity in ABCD

ABCD				
**Age**	**NCT connectivity**	**Temperature**	**Beta**	**connectivity@t1 * beta@tn**
10.50	11.68	1.00	1.00	11.68
11.00	13.35	0.87	1.15	13.47
11.50	13.51	0.86	1.17	13.62
12.00	14.33	0.80	1.24	14.52
12.50	15.35	0.75	1.33	15.50
13.00	14.80	0.77	1.30	15.13
13.50	16.48	0.69	1.44	16.83
14.00	18.77	0.60	1.66	19.38

Table S27 | Connectivity in ALSPAC

ALSPAC				
**Age**	**NCT connectivity**	**Temperature**	**Beta**	**connectivity@t1 * beta@tn**
11	34.07	1.00	1.00	34.07
13	38.23	0.89	1.13	38.38
14	38.78	0.86	1.16	39.48
17	43.30	0.78	1.28	43.64
18	42.74	0.75	1.34	45.67
19	44.20	0.70	1.43	48.73

Table S28 | Connectivity in MCS

MCS				
**Age**	**NCT connectivity**	**Temperature**	**Beta**	**connectivity@t1 * beta@tn**
11	9.61	1.00	1.00	9.61
14	10.62	0.90	1.11	10.64
17	12.69	0.75	1.33	12.82

12. References

1. Volkow, N. D. *et al.* The conception of the ABCD study: From substance use to a broad NIH collaboration. *Developmental Cognitive Neuroscience* **32**, 4–7 (2018).
2. Jernigan, T. L., Brown, S. A. & Dowling, G. J. The Adolescent Brain Cognitive Development Study. *J Res Adolesc* **28**, 154–156 (2018).
3. Achenbach, T., McConaughy, S., Ivanova, M. & Rescorla, L. Manual for the ASEBA brief problem monitor (BPM). (2011).
4. Piper, B. J., Gray, H. M., Raber, J. & Birkett, M. A. Reliability and validity of Brief Problem Monitor, an abbreviated form of the Child Behavior Checklist. *Psychiatry Clin Neurosci* **68**, 759–767 (2014).
5. Richter, J. Preliminary evidence for good psychometric properties of the Norwegian version of the Brief Problems Monitor (BPM). *Nord J Psychiatry* **69**, 174–178 (2015).
6. Rognstad, K., Helland, S. S., Neumer, S.-P., Baardstu, S. & Kjøbli, J. Short measures of youth psychopathology: psychometric properties of the brief problem monitor (BPM) and the behavior and feelings survey (BFS) in a Norwegian clinical sample. *BMC Psychology* **10**, 182 (2022).
7. Fraser, A. *et al.* Cohort Profile: the Avon Longitudinal Study of Parents and Children: ALSPAC mothers cohort. *Int J Epidemiol* **42**, 97–110 (2013).
8. Boyd, A. *et al.* Cohort Profile: the ’children of the 90s’--the index offspring of the Avon Longitudinal Study of Parents and Children. *Int J Epidemiol* **42**, 111–127 (2013).

9. Thapar, A. & McGuffin, P. Validity of the shortened Mood and Feelings Questionnaire in a community sample of children and adolescents: a preliminary research note. *Psychiatry Research* **81**, 259–268 (1998).
10. Turner, N., Joinson, C., Peters, T. J., Wiles, N. & Lewis, G. Validity of the Short Mood and Feelings Questionnaire in late adolescence. *Psychological Assessment* **26**, 752–762 (2014).
11. Connelly, R. & Platt, L. Cohort Profile: UK Millennium Cohort Study (MCS). *International Journal of Epidemiology* **43**, 1719–1725 (2014).
12. Joshi, H. E. & Fitzsimons, E. The UK Millennium Cohort: the making of a multipurpose resource for social science and policy. *Longitudinal and Life Course Studies* **7**, 409–430 (2016).
13. Bryant, A., Guy, J., Team, T. C. & Holmes, J. The Strengths and Difficulties Questionnaire Predicts Concurrent Mental Health Difficulties in a Transdiagnostic Sample of Struggling Learners. *Frontiers in Psychology* **11**, (2020).
14. Armitage, J. M. *et al.* Validation of the Strengths and Difficulties Questionnaire (SDQ) emotional subscale in assessing depression and anxiety across development. *PLOS ONE* **18**, e0288882 (2023).
