# Supplementary material for: Network temperature as a metric of stability in depression symptoms across adolescence
Source: Nat Ment Health. 2025 Apr 29;3(5):548–57. doi: 10.1038/s44220-025-00415-5 (PMC12066352; doi:10.1038/s44220-025-00415-5)
Supplement: Supplementary file 2 — Reporting Summary [file 44220_2025_415_MOESM2_ESM.pdf]

nature portfolio

Corresponding author(s): Poppy Z Grimes
Last updated by author(s): Mar 4, 2025

Reporting Summary

Nature Portfolio wishes to improve the reproducibility of the work that we publish. This form provides structure for consistency and transparency in reporting. For further information on Nature Portfolio policies, see our Editorial Policies and the Editorial Policy Checklist.

Statistics

For all statistical analyses, confirm that the following items are present in the figure legend, table legend, main text, or Methods section.

n/a	Confirmed	
☐	☒	The exact sample size (n) for each experimental group/condition, given as a discrete number and unit of measurement
☐	☒	A statement on whether measurements were taken from distinct samples or whether the same sample was measured repeatedly
☐	☒	The statistical test(s) used AND whether they are one- or two-sided<br>*Only common tests should be described solely by name; describe more complex techniques in the Methods section.*
☐	☒	A description of all covariates tested
☐	☒	A description of any assumptions or corrections, such as tests of normality and adjustment for multiple comparisons
☐	☒	A full description of the statistical parameters including central tendency (e.g. means) or other basic estimates (e.g. regression coefficient) AND variation (e.g. standard deviation) or associated estimates of uncertainty (e.g. confidence intervals)
☐	☒	For null hypothesis testing, the test statistic (e.g. F, t, r) with confidence intervals, effect sizes, degrees of freedom and P value noted<br>*Give P values as exact values whenever suitable.*
☒	☐	For Bayesian analysis, information on the choice of priors and Markov chain Monte Carlo settings
☒	☐	For hierarchical and complex designs, identification of the appropriate level for tests and full reporting of outcomes
☒	☐	Estimates of effect sizes (e.g. Cohen's d, Pearson's r), indicating how they were calculated

Our web collection on statistics for biologists contains articles on many of the points above.

Software and code

Policy information about availability of computer code

Data collection	No software was used
Data analysis	Analyses were performed in R 4.4.1. We used the following R packages: MICE (3.16.0), psychonetrics (0.13), NetworkComparisonTest (v2.2.2), qgraph (1.9.8), IsingSampler(0.2.3). All code and analysis scripts are available on GitHub: https://github.com/poppyzenzi/network_temperature.

For manuscripts utilizing custom algorithms or software that are central to the research but not yet described in published literature, software must be made available to editors and reviewers. We strongly encourage code deposition in a community repository (e.g. GitHub). See the Nature Portfolio guidelines for submitting code & software for further information.

Data

Policy information about availability of data

All manuscripts must include a data availability statement. This statement should provide the following information, where applicable:

- Accession codes, unique identifiers, or web links for publicly available datasets
- A description of any restrictions on data availability
- For clinical datasets or third party data, please ensure that the statement adheres to our policy

ALSPAC: The ALSPAC study website contains details of all the data that are available through a fully searchable data dictionary at http://www.bristol.ac.uk/alspac/researchers/access/. Permission to use the ALSPAC data is obtained through a proposal system managed by the ALSPAC executive.

ABCD: Data used in the preparation of this article were obtained from the Adolescent Brain Cognitive Development (ABCD) Study (https://abcdstudy.org), held in the NIMH Data Archive (NDA). The ABCD data repository grows and changes over time. The ABCD data used in this report came from https://dx.doi.org/10.15154/8873-zj65. DOIs can be found at https://nda.nih.gov/abcd/.
MCS: Millennium Cohort Study data are freely available and can be downloaded on the UK Data Service website (https://ukdataservice.ac.uk/).

Research involving human participants, their data, or biological material

Policy information about studies with human participants or human data. See also policy information about sex, gender (identity/presentation), and sexual orientation and race, ethnicity and racism.

Reporting on sex and gender	We use the term sex, as provided by the studies themselves. Overall numbers are presented in the manuscript for percentage female at each wave of data for each study.
Reporting on race, ethnicity, or other socially relevant groupings	We use the terms as provided by the studies themselves. ALSPAC and MCS are White European cohorts. ABCD is an ethnically diverse cohort and has the following categories of self-reported ethnicity: Asian, Black, Hispanic, White and Other.
Population characteristics	ALSPAC: Pregnant women resident in Avon, UK with expected dates of delivery between 1st April 1991 and 31st December 1992 were invited to take part in the study. The initial number of pregnancies enrolled was 14,541 with 13,988 children who were alive at 1 year of age. The total sample size for analyses using any data collected after the age of seven is therefore 15,447 pregnancies, resulting in 15,658 foetuses. Of these 14,901 children were alive at 1 year of age. In ALSPAC, we used the Short Mood and Feelings Questionnaire (SMFQ; 13 items) collected at 6 waves between ages 11 and 19 years. 55% of participants were female on average across the waves. ALSPAC is a White European cohort.<br>ABCD: The Adolescent Brain and Cognitive Development (ABCD) study cohort has recruited a total number of 11,876 children starting in 2015 across 21 different North American study sites. In ABCD, the self-report depression symptoms were acquired from the internalising subscale of the self-report Brief Problem Monitoring scale (BPM; 6 items) collected at 8 waves between 10 and 14 years. 47% of participants were female on average across the waves. ABCD is an ethnically diverse cohort and observed self-reported ethnicity of 250 Asian, 1743 Black, 2364 Hispanic, 6134 White and 1234 Other individuals.<br>MCS: The Millennium Cohort Study (MCS) which began in 2000 (birth year of participants between 2000-02), is conducted by the Centre for Longitudinal Studies (CLS). It aims to chart the conditions of social, economic and health advantages and disadvantages facing children born at the start of the 21st century The study has been tracking the 'Millennium children' through their early childhood years and plans to follow them into adulthood. In MCS, we used the emotional problems subscale from the parent-report Strength and Difficulties Questionnaire (SDQ; 5 items) collected at 3 waves between ages 11 and 17 years. 51% of participants were female on average across the waves. MCS is a White European cohort.
Recruitment	Recruitment from each study can be found in the corresponding ABCD, ALSPAC and MCS cohort study websites.
Ethics oversight	ABCD: The study was approved by the National Institute of Mental Health Data Archive, United States (NIMH). Written consent was obtained from all participants.<br>ALSPAC: Ethical approval for the study was obtained from the ALSPAC Ethics and Law Committee and the Local Research Ethics Committees. Consent for biological samples has been collected in accordance with the Human Tissue Act (2004). Informed consent for the use of data collected via questionnaires and clinics was obtained from participants following the recommendations of the ALSPAC Ethics and Law Committee at the time.<br>MCS:The MCS was approved by the South West and London Multi-Centre Research Ethics Committees. The Millennium Cohort Study obtained informed written consent from parent/ guardians of the cohort children in order to participate in the study, children themselves as they grow-up and other participants as necessary.

Note that full information on the approval of the study protocol must also be provided in the manuscript.

Field-specific reporting

Please select the one below that is the best fit for your research. If you are not sure, read the appropriate sections before making your selection.

☐ Life sciences ☒ Behavioural & social sciences ☐ Ecological, evolutionary & environmental sciences

For a reference copy of the document with all sections, see nature.com/documents/nr-reporting-summary-flat.pdf

Behavioural & social sciences study design

All studies must disclose on these points even when the disclosure is negative.

Study description	This is a quantitative study that uses network analysis with longitudinal cohort data
Research sample	Participants were from the ABCD, ALSPAC and MCS longitudinal cohort studies (existing datasets).<br>ALSPAC: Pregnant women resident in Avon, UK with expected dates of delivery between 1st April 1991 and 31st December 1992 were invited to take part in the study. The initial number of pregnancies enrolled was 14,541 with 13,988 children who were alive at 1 year of age. The total sample size for analyses using any data collected after the age of seven is therefore 15,447 pregnancies, resulting in 15,658 foetuses. Of these 14,901 children were alive at 1 year of age. In ALSPAC, we used the Short Mood and Feelings Questionnaire (SMFQ; 13 items) collected at 6 waves between ages 11 and 19 years. 55% of participants were female on average across the waves. ALSPAC is a White European cohort. The ALSPAC study website contains details of all the data that are available through a fully searchable data dictionary at http://www.bristol.ac.uk/alspac/researchers/access/. Permission to use the ALSPAC data

is obtained through a proposal system managed by the ALSPAC executive.

ABCD: The Adolescent Brain and Cognitive Development (ABCD) study cohort has recruited a total number of 11,876 children starting in 2015 across 21 different North American study sites. In ABCD, the self-report depression symptoms were acquired from the internalising subscale of the self-report Brief Problem Monitoring scale (BPM; 6 items) collected at 8 waves between 10 and 14 years. 47% of participants were female on average across the waves. ALSPAC is a White European cohort. ABCD is an ethnically diverse cohort and observed self-reported ethnicity of 250 Asian, 1743 Black, 2364 Hispanic, 6134 White and 1234 Other individuals. Data used in the preparation of this article were obtained from the Adolescent Brain Cognitive Development (ABCD) Study (https://abcdstudy.org), held in the NIMH Data Archive (NDA). The ABCD data repository grows and changes over time. The ABCD data used in this report came from https://dx.doi.org/10.15154/8873-zj65. DOIs can be found at https://nda.nih.gov/abcd/.

MCS: The Millennium Cohort Study (MCS) which began in 2000 (birth year of participants between 2000-02), is conducted by the Centre for Longitudinal Studies (CLS). It aims to chart the conditions of social, economic and health advantages and disadvantages facing children born at the start of the 21st century The study has been tracking the 'Millennium children' through their early childhood years and plans to follow them into adulthood. In MCS, we used the emotional problems subscale from the parent-report Strength and Difficulties Questionnaire (SDQ; 5 items) collected at 3 waves between ages 11 and 17 years. 51% of participants were female on average across the waves. MCS is a White European cohort. Millennium Cohort Study data are freely available and can be downloaded on the UK Data Service website (https://ukdataservice.ac.uk/).

Sampling strategy

ALSPAC: Participants were recruited from a geographically defined region in the UK, where pregnant women were invited to join the study. The cohort was primarily composed of mothers living in the Avon area who were due to give birth between 1991 and 1992.
ABCD: Participants were selected through stratified random sampling from across the United States, ensuring representation across diverse demographic characteristics, such as race, ethnicity, and socio-economic status.
MCS: Participants were selected using stratified random sampling from across the UK, ensuring a nationally representative sample that accounted for geographical regions, social class, and ethnicity.

Data collection

Data collection varied between cohorts. See the individual cohorts themselves. Briefly, ALSPAC used the Short Mood and Feelings questionnaire (SMFQ), ABCD used the Brief Problem Monitoring (BPM) scale and MCS used the Strength and Difficulties Questionnaire (SDQ). The SMFQ is a 13-item questionnaire that measures the presence of depressive symptoms in the last two weeks, and has a clinical cut-off as a total score of >=11. The SMFQ has been clinically validated against ICD-10 depression diagnosis. The Brief Problem Monitoring (internalising) is a 6-symptom subscale with items derived using factor analysis and item response theory from CBCL and YSR. BPM has a clinical T-score cut off of >65 (from the ASEBA manual). Internal consistency and correspondence with CBCL has been demonstrated. The SDQ is a brief behavioural screening questionnaire about 2- to 17-year- olds. The emotional symptoms subscale is used to measure information about mood and feelings related to depression. This subscale has a clinical cut-off of >= 5 for depression status. The researcher was not blinded to the study hypothesis or experimental condition, as the study aimed to analyze existing data from cohort studies.

Timing

ABCD: 09-2015 - ongoing, ALSPAC: 04-1991 - ongoing, MCS: 09-2000 - ongoing.

Data exclusions

No data were excluded from these analyses

Non-participation

All participants are included in these analyses

Randomization

Participants were not allocated into experimental groups, as this study used existing data from observational cohort studies (ALSPAC, ABCD, MCS). There was no randomisation. This was a longitudinal investigation so age was not included as a covariate. Sex was stratified for one analysis as part of the investigation.

Reporting for specific materials, systems and methods

We require information from authors about some types of materials, experimental systems and methods used in many studies. Here, indicate whether each material, system or method listed is relevant to your study. If you are not sure if a list item applies to your research, read the appropriate section before selecting a response.

Materials & experimental systems

n/a	Involved in the study
☒	☐ Antibodies
☒	☐ Eukaryotic cell lines
☒	☐ Palaeontology and archaeology
☒	☐ Animals and other organisms
☒	☐ Clinical data
☒	☐ Dual use research of concern
☒	☐ Plants

Methods

n/a	Involved in the study
☒	☐ ChIP-seq
☒	☐ Flow cytometry
☒	☐ MRI-based neuroimaging

Plants

Seed stocks	*Report on the source of all seed stocks or other plant material used. If applicable, state the seed stock centre and catalogue number. If plant specimens were collected from the field, describe the collection location, date and sampling procedures.*
Novel plant genotypes	*Describe the methods by which all novel plant genotypes were produced. This includes those generated by transgenic approaches, gene editing, chemical/radiation-based mutagenesis and hybridization. For transgenic lines, describe the transformation method, the number of independent lines analyzed and the generation upon which experiments were performed. For gene-edited lines, describe the editor used, the endogenous sequence targeted for editing, the targeting guide RNA sequence (if applicable) and how the editor was applied.*
Authentication	*Describe any authentication procedures for each seed stock used or novel genotype generated. Describe any experiments used to assess the effect of a mutation and, where applicable, how potential secondary effects (e.g. second site T-DNA insertions, mosiacism, off-target gene editing) were examined.*
